# Hyper-Crosslinked Polymer Nanocomposites Containing Mesoporous Silica Nanoparticles with Enhanced Adsorption Towards Polar Dyes

**DOI:** 10.3390/polym12061388

**Published:** 2020-06-20

**Authors:** Marco Guerritore, Rachele Castaldo, Brigida Silvestri, Roberto Avolio, Mariacristina Cocca, Maria Emanuela Errico, Maurizio Avella, Gennaro Gentile, Veronica Ambrogi

**Affiliations:** 1 Department of Chemical, Materials and Production Engineering (DICMaPI)—University of Naples Federico II, P. le Tecchio 80, 80125 Napoli, Italy; marco.g788@gmail.com (M.G.); ambrogi@unina.it (V.A.); 2 National Research Council of Italy, Institute for Polymers Composites and Biomaterials (IPCB-CNR), Via Campi Flegrei 34, 80078 Pozzuoli (NA), Italy; roberto.avolio@cnr.it (R.A.); mariacristina.cocca@cnr.it (M.C.); mariaemanuela.errico@cnr.it (M.E.E.); maurizio.avella@cnr.it (M.A.); gennaro.gentile@cnr.it (G.G.)

**Keywords:** hyper-crosslinked resins, mesoporous silica nanoparticles, adsorption, dyes

## Abstract

The development of new styrene-based hyper-crosslinked nanocomposites (HCLN) containing mesoporous silica nanoparticles (MSN) is reported here as a new strategy to obtain functional high surface area materials with an enhanced hydrophilic character. The HCLN composition, morphology and porous structure were analyzed using a multi-technique approach. The HCLN displayed a high surface area (above 1600 m^2^/g) and higher microporosity than the corresponding hyper-crosslinked neat resin. The enhanced adsorption properties of the HCLN towards polar organic dyes was demonstrated through the adsorption of a reactive dye, Remazol Brilliant Blue R (RB). In particular, the HCLN containing 5phr MSN showed the highest adsorption capacity of RB.

## 1. Introduction

Water pollution is a very relevant and complex worldwide environmental issue. The complexity of this phenomenon mainly depends on the great variety of pollutants that foul up the aquatic environments. To face this issue, each source of pollution must be addressed in a very specific way.

Synthetic dyes, in particular, are a category of pollutants that are very dangerous for the environment, because they have been demonstrated to be very persistent to degradation and, even at low concentration, they significantly prevent the use, or reuse, of water. The removal of these contaminants is therefore of paramount importance [1]. Many approaches have been pursued for the decontamination of polluted water from synthetic dyes, including adsorption, photocatalytic degradation, biodegradation, coagulation, flocculation and electrolysis [2,3,4,5,6,7]. Among them, physical adsorption is one of the most advantageous strategies, due to the spontaneity, reversibility and low energetic impact of the process [8].

Out of the variety of adsorbent materials, hyper-crosslinked resins (HCLR) are microporous/mesoporous polymers that can display very different pore size distributions and specific surface areas (SSA) [9]. This class of polymers can easily be functionalized to promote the adsorption of specific pollutants [10,11,12,13]. In particular, high surface area styrene-based HCLR were recently obtained by a facile high yield synthesis based on the bulk polymerization of a precursor polymer, followed by Friedel–Crafts alkylation. A HCLR characterized by a specific surface area of about 2000 m^2^/g, micro and mesoporous texture, and high thermal and chemical resistance was developed [14]. Moreover, through this procedure, the embedding and dispersion of different inorganic nanofillers was exploited as a successful strategy to tune specific properties of the resin. Several HCLR-based nanocomposites have been synthesized [15], either to modify the textural or adsorption properties of the resin [14,16], or to transfer to the high surface area resin specific features of the nanofillers, such as catalytic activity [17,18] or magnetic properties [19].

In this work, novel nanocomposite materials based on HCLR and mesoporous silica nanoparticles (MSN) were developed with the aim to obtain a new porous system with increased adsorption capacity towards synthetic dyes. MSN, with their highly controllable pore size, geometry and surface properties, as well as their low cost, are one of the most versatile adsorbents for the removal of contaminants from water [20,21,22]. Indeed, the sol–gel methodology allows a fine tuning of their morphology [23], thus influencing the final properties of the systems [24,25,26,27]. Moreover, MSN can be easily functionalized in order to improve their adsorption properties [28]. 

Here, for the first time, the MSN surface hydroxyl functionalities are exploited to confer a more polar character to the HCLR. At the same time, the MSN porosity is expected to be interconnected with the HCLR micro and mesoporosity, constituting together an extended porous network, ultimately leading to an enhanced adsorption capacity towards polar dyes. With this aim, monodispersed high SSA mesoporous silica nanoparticles were synthesized using a surfactant-assisted sol–gel technique. Then, hyper-crosslinked resins and nanocomposites containing MSN were prepared through a facile synthetic approach based on the bulk polymerization of a gel-type precursor and its hyper-crosslinking through a Friedel–Crafts reaction. The nanocomposite materials were widely characterized from the physical, chemical and morphological point of view, and adsorption tests were performed to prove their efficiency as adsorbents for polar organic pollutants. Considering the high concentration of reactive dyes found in aquatic environments, due to their frequent use in the textile industry (reactive dyes constitute about 45% of dyes produced for the textile industry), a reactive dye, Remazol Brilliant Blue R, was chosen as the model dye for the adsorption experiments [29].

## 2. Materials and Methods 

### 2.1. Materials

Vinylbenzyl chloride (VBC, ≥95.0%, mixture of isomers, ~70% meta + ~30% para), p-divinylbenzene (DVB, 85%, meta isomer ~10 wt%), 2,2′-azobis (2-methylpropionitrile) (AIBN, >98%), tetraethyl orthosilicate (TEOS, 99.999%), cetyltrimethylammonium bromide (CTAB, ≥99%), 1,2-dichloroethane (DCE), Remazol Brilliant Blue R (RB), hydrochloric acid (HCl, 37 wt%), NaOH (≥97.0%, pellets), FeCl_3_ (≥97%) and all solvents (ethanol, methanol and acetone) were purchased from Sigma-Aldrich (Milan, Italy) and used without further purification. 

### 2.2. Synthesis of Mesoporous Silica Nanoparticles

MSN were prepared following a previously reported sol–gel procedure [30]. Briefly, 1.0 g CTAB was dissolved in 480 mL distilled water, then a sodium hydroxide aqueous solution (2.00 M, 3.50 mL) was introduced into the CTAB solution, and the temperature of the mixture was adjusted to 80 °C. Therefore, TEOS (5.00 mL) was added dropwise to the surfactant solution under vigorous stirring, and the mixture was allowed to react for 2.5 h. The obtained product was transferred into centrifuge tubes and centrifuged at 11,000 rpm for 10 min at 10 °C. Then, the supernatant was removed and the MSN were washed twice with ethanol and centrifuged again under the same conditions. Finally, they were refluxed three times in an HCl (37%)/ethanol solution at 1/8 vol/vol, and ultimately suspended in ethanol.

### 2.3. Synthesis of Hyper-Crosslinked Resins and Nanocomposites 

Hyper-crosslinked resins and nanocomposites containing MSN were prepared by a two-step procedure. DVB and VBC were mixed in a vial in the molar composition 2:98, then 0.5 phr AIBN were added and the mixture was stirred for 30 min under nitrogen. Then, the vial was kept in an oven at 80 °C for 24 h. The obtained polymer was coded DV [14]. 

The nanocomposites were prepared using the same DVB/VBC molar composition of the resin with an added 5 or 15 phr MSN, which were previously dried from the ethanol suspension. Next, the DVB/VBC/MSN mixtures were sonicated for 30 min with a 500 W tip sonicator at 25% power, with a 10 s/50 s ON/OFF cycle. Then, 0.5 phr AIBN was added, and the mixtures were stirred at 80 °C under nitrogen flux. This stage was prolonged until the mixture’s viscosity increased enough to hinder nanoparticles re-aggregation, in each case for about 30 min, and then the polymerization was completed in an oven for 24 h in a closed vial. The obtained nanocomposites were coded DV-xMSN, where x is the nominal content of MSN. Resin and nanocomposites were washed with methanol and acetone and dried overnight under vacuum at 50 °C.

Next, the precursor resin and nanocomposites were swollen in DCE, under nitrogen, for 2 h. Then, they were cooled to 0 °C in an ice/water bath, and the Friedel–Crafts catalyst, FeCl_3_, was added. The system was heated to 80 °C and kept at this temperature for 18 h. The as-obtained hyper-crosslinked resins and nanocomposites were washed repeatedly with methanol and dried under vacuum at 80 °C overnight. Hyper-crosslinked samples were coded as the corresponding precursor resins and nanocomposites by adding the prefix X-.

### 2.4. Characterization

MSN were analyzed by Fourier Transformed Infrared (FTIR) spectroscopy. FTIR spectra were recorded in the 4000−400 cm^−1^ range, using a Nexus FT IR spectrometer equipped with a DTGS KBr (deuterated triglycine sulfate with potassium bromide windows) detector. The spectral resolution was 2 cm^−1,^ and each spectrum represents an average of 64 scans. Samples for FTIR analysis were prepared by pressing a mixture of KBr and dried MSN (2.0 wt%) into 13 mm diameter disks. 

All the precursors and hyper-crosslinked resin and nanocomposites were analyzed by FTIR in total attenuated reflection (ATR) mode. Spectra were recorded with a PerkinElmer Spectrum One FTIR spectrometer equipped with an ATR module, using a resolution of 4 cm^−1^ and 32 scan collections.

Precursors, hyper-crosslinked resins and nanocomposites were analyzed by solid-state ^13^C CP-MAS NMR, using a Bruker Avance II 400 spectrometer equipped with a 4 mm magic angle spinning (MAS) probe. Samples were packed in 4 mm zirconia rotors sealed with Kel-F caps and rotated at a spinning speed between 11 and 13 kHz. A ^1^H π/2 pulse width of 3.6 μs, a contact time of 2 ms and a repetition time of 5 s were used. 

Bright field transmission electron microscopy (TEM) analysis was performed on MSN using a FEI Tecnai G12 Spirit Twin (LaB_6_ source) at 120 kV acceleration voltage. Images were collected on a FEI Eagle 4k CCD camera. Before the analysis, MSN were dispersed in ethanol by ultrasonication for 2 min with a 500 W tip sonicator set at 25% power. The samples were then collected by immersing holey carbon-coated copper grids in the dispersions. The size distribution of MSN nanoparticles was determined through image analysis using the software ImageJ. About 400 MSN nanoparticles were measured, and the average size, standard deviation and log-normal distribution curve were obtained.

The hyper-crosslinked nanocomposites were characterized by SEM analysis to evaluate the morphology and dispersion of MSN within the precursor resins. The analysis was performed using a FEI Quanta 200 FEG SEM. A small amount of hyper-crosslinked nanocomposites were deposited on aluminum stubs covered with carbon adhesive disks, and the analysis was performed using a secondary electron detector and an acceleration voltage of 10–30 kV.

Thermogravimetric analysis was carried out on MSN, XDV and XDV-xMSN using a Perkin Elmer Pyris Diamond thermogravimetric analyzer. All the samples were analyzed in an oxidizing atmosphere, at 10 °C/min heating rate, from room temperature to 800 °C.

Gas adsorption volumetric analysis was performed on MSN and on the hyper-crosslinked resin and nanocomposites using a Micromeritics ASAP 2020 analyzer. SSA was determined by nitrogen adsorption measurements at 77 K from the linear part of the Brunauer–Emmett–Teller (BET) equation. Nonlocal density functional theory (NLDFT) and the Barrett–Joyner–Halenda (BJH) method were applied to the nitrogen adsorption isotherms to evaluate the pore size distribution of the materials. A cylindrical pore model was applied for MSN, and a slit pore model was applied for HCLR and HCLN NLDFT pore size distribution. The micropore fraction of the hyper-crosslinked resin and nanocomposites was evaluated as the percentage fraction between the microporous volume and the total volume evaluated by NLDFT. Prior to the analysis, the MSN were degassed at 200 °C, and the resin and nanocomposites were degassed at 120 °C under vacuum (*p* < 10^−5^ mbar). All the adsorption measurements were performed using high purity gases (>99.999%). 

Finally, MSN, hyper-crosslinked resin and nanocomposites were tested for the adsorption of a reactive dye from water, namely Remazol Brilliant Blue R. Tests were performed at 25 °C at various dye concentrations ranging from 10 to 500 mg/L. Samples of about 10 mg were introduced into vials containing 10 mL of dye solution, and the vials were kept at 25 °C until equilibrium was reached (at least 48 h). The supernatant dye concentration was measured by means of a Jasco V570 UV spectrophotometer, using a RB previously recorded calibration curve. The amount of dye adsorbed (*Q_e_*) was determined as

(1) $$Q_{e}=\frac{\left(C_{0}-C_{f}\right)V}{m}$$

where *C*_0_ and *C_f_* are the initial and final concentration of the dye solution, *V* is the volume of dye solution and *m* is the mass of the adsorbent.

## 3. Results

### 3.1. Mesoporous Silica Nanoparticles

**Figure 1:**
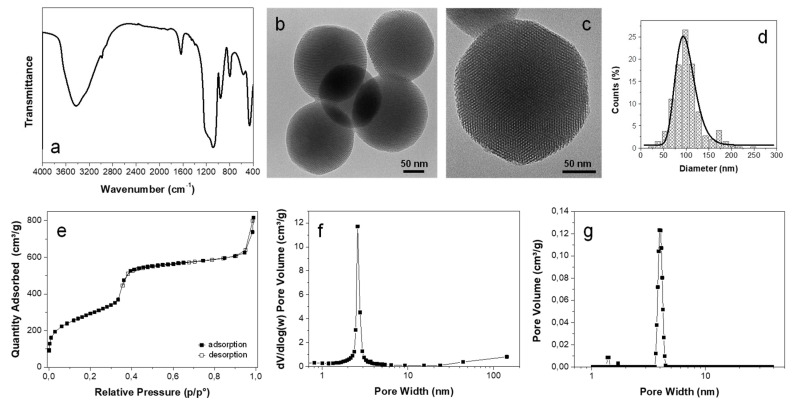
MSN FTIR spectrum (**a**) TEM images (**b**,**c**), particle size distribution (**d**), nitrogen adsorption isotherm (**e**), and pore size distribution by BJH (**f**) and NLDFT (**g**) models.

MSN were successfully obtained through a modified sol–gel synthesis, using CTAB as a templating agent. The yield of the reaction was about 65%. FTIR spectroscopy (Figure 1a) revealed, for the MSN, the characteristic silica absorption peaks. In particular, the peak at 459 cm^−1^ is attributed to the bending vibration of O–Si–O, the strong absorption band at 1086 cm^−1^ corresponds to the Si–O–Si stretching vibration, the peak at 798 cm^−1^ corresponds to the silicon ‘cage’ motion, the peaks at 960 cm^−1^ and 1639 cm^−1^ correspond to the Si–OH bending vibration and the broad absorption band at 3421 cm^−1^ is attributed to the O–H stretching vibration [31]. The intensity of the signals attributed to the O-H vibration modes indicate that the synthesized MSN are characterized by a large number of surface hydroxyl groups [32].

MSN morphology was observed by TEM analysis. As shown in Figure 1b,c, the particles show an almost perfectly spherical shape and the presence of ordered porous channels characteristic of mesoporous silica. MSN are characterized by an average diameter of 102 ± 32 nm. It is worthwhile to note that the size distribution of the nanoparticles is quite homogeneous, since 75% of the nanoparticles are in the range 70–130 nm (see Figure 1d).

The N_2_ adsorption–desorption isotherm of MSN, reported in Figure 1e, is a type IV isotherm [33] which is typical of mesoporous silica. The adsorption–desorption curve is of sigmoidal shape, with a sharp inflection between 0.3 and 0.4 p/p^0^, which reveals the uniformity of the pore size distribution. The specific surface area of the MSN, as evaluated by a BET model, is of 1050 ± 5 m^2^/g, while the specific pore volume of the MSN, as evaluated by the NLDFT model, is 0.83 cm^3^/g. BJH and NLDFT pore size distributions, shown in Figure 1f,g, also show a monodisperse pore size distribution of around 2.6 nm and 3.6 nm, respectively. The difference in the average pore size detected by the two models is expected, since BJH is reported to possibly underestimate pore size and to not treat realistically the behaviour of the molecules adsorbed in pore structures [34]. Nevertheless, BJH is largely used for mesoporous materials, while it is not appropriate for the micropore size range. Therefore, the BJH model is very useful to allow comparison with the wide literature on mesoporous silica nanoparticles [30,35], while the use of the NLDFT model provides a more realistic estimation of MSN porosity, and also allows direct comparison between the pore size distribution of the MSN and the nanocomposite systems developed. It is worthwhile to note that the average pore size derived by the NLDFT model is also in line with the value obtained by measuring the dimension of the pore channels observed in TEM images, which is about 3.8 ± 0.4 nm.

### 3.2. Hyper-Crosslinked Resins and Nanocomposites

Hyper-crosslinked resins and nanocomposites were prepared by an optimized synthetic procedure already detailed elsewhere [14]. This protocol was based on the bulk polymerization of a precursor polymer, followed by hyper-crosslinking through a Friedel–Crafts reaction. 

The MSN were added to the monomeric mixture at a 5 and 15 phr loading. The bulk polymerization was performed, and its yield (99% for DV) decreased to 95% and 80% for DV-5MSN and DV-15MSN, respectively. FTIR analysis was performed on the residues of the washing solutions, and confirmed that no silica nanoparticles were lost during the washing of the precursor nanocomposites. Therefore, the yield decrease is to be attributed to a certain amount of residual oligomeric fractions. Indeed, the inclusion of MSN induces the increase of the viscosity of the system, which could partially obstruct the progress of the polymerization reaction due to a reduced mobility of the growing polymer fraction at the late stage of polymerization. As a consequence of the decrease of the organic phase content, the actual content of MSN in the final nanocomposite was re-calculated, resulting in 5.4 phr and 19.0 phr for DV-5MSN and DV-15MSN, respectively.

**Figure 2:**
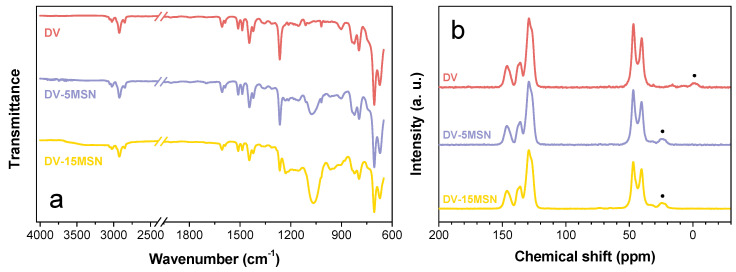
FTIR (**a**) and NMR (**b**) spectra of DV and DV-xMSN. Spinning sidebands are marked by dots in (**b**).

Precursor resin DV and nanocomposites DV-xMSN were analyzed by means of a FTIR in ATR mode. All spectra show the characteristic absorption bands of both MSN and DV (Figure 2a). In particular, DV-5MSN and DV-15MSN FTIR spectra clearly show the C–H stretching absorption bands in the range 2950–2850 cm^−1^, the DV fingerprint signals in the range 1500–500 cm^−1^ and the silica strong absorption band at 1086 cm^−1^, corresponding to the Si–O–Si stretching vibration. 

NMR analysis confirmed that the presence of the nanofillers, although reducing the yield, showed a negligible influence on the reaction products. Indeed, the ^13^C CP/MAS NMR spectra of DV-x5MSN and DV-x15MSN, reported in Figure 2b, show the typical signals of DV.

**Figure 3:**
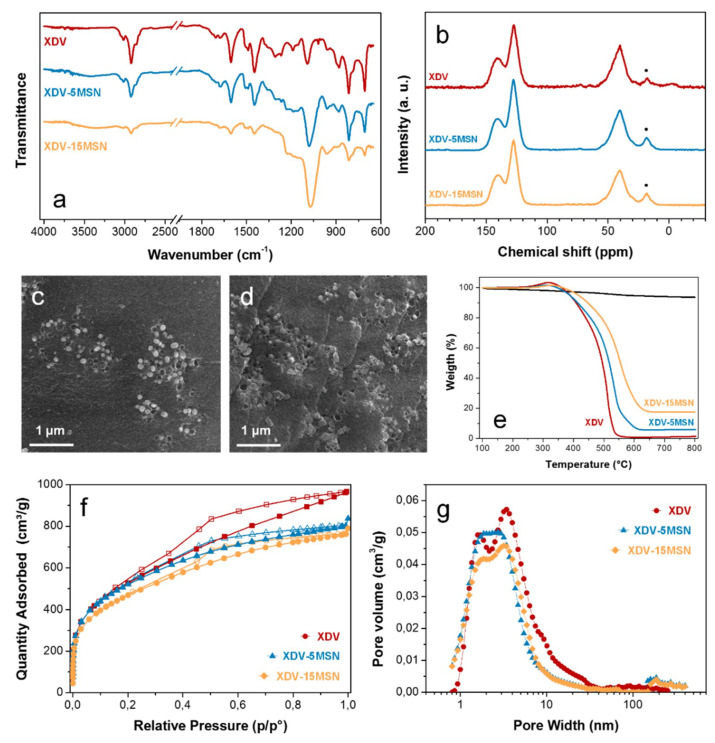
FTIR (**a**) and NMR (**b**, spinning sidebands are marked by dots) spectra of XDV and XDV-xMSN, SEM images of XDV-5MSN and XDV-15MSN (**c**,**d**), TGA traces of XDV and XDV-xMSN (**e**), nitrogen adsorption (full symbols) and desorption (empty symbols) isotherms (**f**), and NLDFT pore size distribution (**g**) of XDV and XDV-xMSN.

Hyper-crosslinked resin and nanocomposites were analyzed by FTIR spectroscopy. The extent of the hyper-crosslinking reaction was confirmed in all samples through the disappearance of the absorption band centered at 1265 cm^−1^, which is diagnostic for the chloromethyl group (Figure 3a). NMR analysis also confirmed the extent of the reaction, with the nearly complete disappearance of the chloromethyl signal (47 ppm), though a quantitative estimation is hindered by the overlapping of the broad aliphatic peak centered at 40 ppm (Figure 3b). 

SEM observation of hyper-crosslinked nanocomposites surfaces revealed a good distribution of the nanoparticles within the polymer matrix, demonstrating that the synthetic approach pursued allowed the obtaining of an effective dispersion of the inorganic nanofillers into the hyper-crosslinked polymer matrix (see Figure 3c,d). However, a poor particle-HCLR matrix adhesion was also registered, since some holes were evidenced, indicating a partial pull out of the MSN particles occurring during hyper-crosslinking. Nevertheless, this phenomenon, which is attributed to the low compatibility between the hydrophilic MSN and the highly hydrophobic resin, can only occur at the surface of the hyper-crosslinked particles, and it does not affect the final MSN content in the nanocomposites.

The hyper-crosslinked resins and nanocomposites thermal stability was evaluated by thermogravimetric analysis in oxidative conditions. MSN show negligible degradation up to 800 °C, showing about 5% weight loss. TGA analysis revealed that the inclusion of MSN in the hyper-crosslinked nanocomposites confers to them increased thermal resistance, since XDV-5MSN and XDV-15MSN show a degradation temperature of about 24 °C and 40 °C higher than XDV degradation temperature (see Figure 3e). Moreover, the XDV-xMSN residues at 800 °C comply with the nominal composition, confirming that the pull out phenomena evidenced by SEM analysis do not affect the composition of the nanocomposites.

Finally, hyper-crosslinked resins and nanocomposites were analyzed by N_2_ adsorption. The adsorption–desorption isotherms for N_2_ at 77K and NLDFT pore size distributions are shown in Figure 3f,g. SSA and porosity data are reported in Table 1. All isotherms are typical type II curves with hysteresis in the desorption branch [33]. With the inclusion of the MSN, the nanocomposite BET SSA and total porosity progressively decrease, as expected, since MSN are characterized by significantly lower SSA than XDV. Therefore, as their content becomes more prominent, their contribution to the surface area and porosity of the nanocomposites does also. On the other hand, the micropore fraction of the nanocomposites significantly increases to 39% and 36% in XDV-5MSN and XDV-15MSN. This result shows that the inclusion of MSN induces the formation of a narrower interconnected network during hyper-crosslinking, resulting in a more microporous structure. 

### 3.3. Equilibrium Adsorption Tests

**Figure 4:**
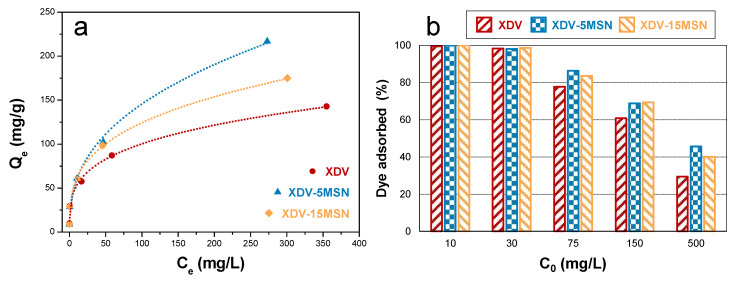
RB equilibrium adsorption (**a**, Freundlich fitting curves are dotted) and the adsorbed dye percentage (**b**) of XDV and XDV-xMSN.

Equilibrium adsorption tests of RB from an aqueous solution in the range of the initial concentration 10–500 mg/L were performed on the hyper-crosslinked resins and nanocomposites. All samples show Freundlich-type adsorption, demonstrating that multilayer adsorption takes place in the porosity of the samples (see Figure 4a and Table 2). Freundlich parameters n and *K_F_* were evaluated. *K_F_* is the Freundlich coefficient indicating the adsorption capacity, while *n* gives an indication of the intensity of the adsorption [36]. The higher the maximum adsorption capacity, the higher the *K_F_* coefficient, and for lower *n* values, the adsorption is more favoured. 

As shown in Figure 4a, by including MSN in the HCLR, the total adsorption capacity of the nanocomposites increases, as well as the slope of the adsorption curves. In fact, *n* decreases in the nanocomposites, in greater form in XDV-5MSN, implying a major adsorption capacity of the nanocomposites at higher dye concentrations. In fact, while all systems show comparable adsorption capacity at the lowest concentrations tested (10 and 30 mg/L), adsorbing about 100% of the dye, at higher concentrations the dye removal efficiency of the hyper-crosslinked resin diminishes, while the nanocomposites are able to remove more dye from water than XDV (see Figure 4b). This result can be attributed to the increased microporosity fraction of the nanocomposites, which drives the adsorption process. Among the nanocomposite systems, with the increasing the amount of MSN from 5 phr to 15 phr, the adsorption of RB partially decreases, and this diminution is correlated to the reduced micropore fraction of XDV-15MSN with respect to XDV-5MSN. These results are particularly relevant if compared with literature data on the most investigated materials for the adsorption of RB, such as activated carbons, which, in the same analysis conditions, show lower adsorption capacity and Langmuir trend [37,38,39]. Moreover, in all cases, the increased adsorption capacity of hyper-crosslinked nanocomposites with respect to XDV is very remarkable, especially if one considers that the total pore volumes of the nanocomposites are lower than those shown by the neat hyper-crosslinked resin, and that pristine MSN show negligible adsorption of RB. Therefore, the inclusion of MSN in a styrene-based hyper-crosslinked resin is a very promising strategy to successfully exploit the high polarity of the hydroxyl functionalities of MSN and their porosity. The inclusion of the MSN in the micro-mesoporous network of the hyper-crosslinked resin phase can indeed be considered a strategy to attract the dye towards the MSN and to exploit their porosity for its adsorption, comparable to the various chemical functionalization strategies developed in the literature [28].

## 4. Conclusions

New hyper-crosslinked nanocomposites based on vinylbenzyl chloride and divinylbenzene, containing mesoporous silica nanoparticles up to 15 phr were developed. The hyper-crosslinked nanocomposites showed high surface area (above 1600 m^2^/g) and a more microporous structure (above 36%) with respect to the neat corresponding HCLR.

For all compositions, the hyper-crosslinked nanocomposites were characterized by increased adsorption capacity towards the tested reactive dye, Remazol Brilliant Blue R, with respect to the neat resin. In particular, the 5 phr MSN composition showed the highest uptake of RB. On the other hand, at 15 phr content, the polymerization of the nanocomposite was partially hindered, with a reduction of the reaction yield to 80%, and the nanocomposite porosity, microporous fraction and adsorption of RB were reduced with respect to the 5 phr MSN composition. Therefore, the 5 phr MSN content represents the optimal composition for the developed hyper-crosslinked nanocomposites, showing high SSA (1824 m^2^/g) and high adsorption of RB.

Overall, the inclusion of MSN in a HCLR has proven to be an efficient strategy to obtain high surface area hyper-crosslinked nanocomposites with interesting textural properties and remarkable adsorption capacity towards a reactive dye at room temperature. 

## Figures and Tables

**Table 1:** BET SSA and porosity of hyper-crosslinked resins and nanocomposites.

Sample	BET SSA (m^2^/g)	Total Pore Volume (cm^3^/g)	Micropore Fraction (%)
XDV	1928 ± 6	1.36	31
XDV-5MSN	1824 ± 23	1.14	39
XDV-15MSN	1644 ± 20	1.08	36

**Table 2:** Freundlich parameters of XDV, XDV-5MSN and XDV-15MSN.

Sample	n	K_F_ [(mg/g)/(mg/L)^1/n^]	R^2^
XDV	3.6	28	0.99
XDV-5MSN	2.6	24.5	0.99
XDV-15MSN	3.3	30.1	0.99
